# Radiographic case report of a heart transplanted patient suffering from COVID-19

**DOI:** 10.1016/j.heliyon.2021.e06688

**Published:** 2021-04-03

**Authors:** Leona S. Alizadeh, Moritz H. Albrecht, Christian Booz

**Affiliations:** a Department of Diagnostic and Interventional Radiology, University Hospital, Frankfurt, Germany; b Department of Diagnostic and Interventional Radiology, Bundeswehrzentralkrankenhaus Koblenz, Koblenz, Germany

**Keywords:** COVID-19, Heart transplantation, Thorax, Multidetector computed tomography

## Abstract

In context of severe acute respiratory syndrome coronavirus 2 (SARS-CoV-2), patients with certain comorbidities and high age, as well as male sex are considered to represent the risk group for severe course of disease. Corona-virus disease 2019 (COVID-19) typical CT-patterns include bilateral, peripheral ground glass opacity (GGO), septal thickening, bronchiectasis, consolidation as well as associated pleural effusion. We report a 77-year-old heart transplanted patient with confirmed COVID-19 infection and coronary heart disease, diabetes type II and other risk factors. Notably, only slight clinical symptoms were reported and repeated computed tomography (CT) scans showed an atypical course of CT findings during his hospitalization.

## 1 Introduction

Informed consent of the patient was obtained for the publication case reports. We report a 77-year-old male patient with an allogeneic heart transplantation (HTX) completed in 2003 and resulting long term immunosuppressive therapy with Sirolimus ([1mg/0,5mg]/d) and Mycophenolat-Mofetil (250mg BID) medication. The patient had risk factors [1] for a severe course of COVID-19 as follows: arterial hypertension, type II diabetes mellitus, and known coronary 2-vessel disease with transplant vasculopathy and mildly restricted left ventricular ejection function. Further, the patient suffered from stage III chronic kidney disease with impaired renal function and hypercholesterinemia.

Initially, the patient presented at our emergency department with an unspecific reduced general condition (slight weight loss of 4kg in 2 weeks, fatigue and stress dyspnea). However, the patient showed no severe respiratory infection indicators or increased body temperature. There was no contact to known positive COVID-19 patients and a negative travel history. The initial blood gas analysis (aBGA) showed a pH of 7.51. In combination with enhanced respiratory frequency (>22/min), the diagnosis of respiratory alkalosis was made and a non-contrast CT scan was performed to rule out an infectious genesis [2]. Later on the same day, the real-time polymerase chain reaction (RT-PCR) testing for COVID-19 yielded a positive result. Subsequently, early antiviral treatment was started. Hydroxychloroquine was given rather than Ritonavir in order to avoid drug interactions which may lead to toxic drug levels, as the immunosuppressive therapy interacts with cytochrome P450 pathways [3, 4, 5].

Under this regimen, the patient improved steadily. The supplementary oxygen initially required could be reduced gradually after day three and was cancelled after seven days. Dyspnea, cough and fatigue improved, and the patient showed no related symptoms after ten days. Inflammation markers and known prognostic markers such as D-Dimers, Troponin, LDH and Ferritin decreased.

## 2 CT findings

During hospitalization, the patient underwent three non-contrast CT scans performed on a 64-slice CT-scanner [SOMATOM Sensation 64 eco; Siemens Healthineers, Forchheim, Germany]. All images were acquired at full inspiration in one single inspiratory breath-hold without intravenous contrast. A 120 kVp protocol, with 500 ms exposure time and a pitch of 1.5 with head first side patient position and no use of radiation filters.

**Figure 1:**
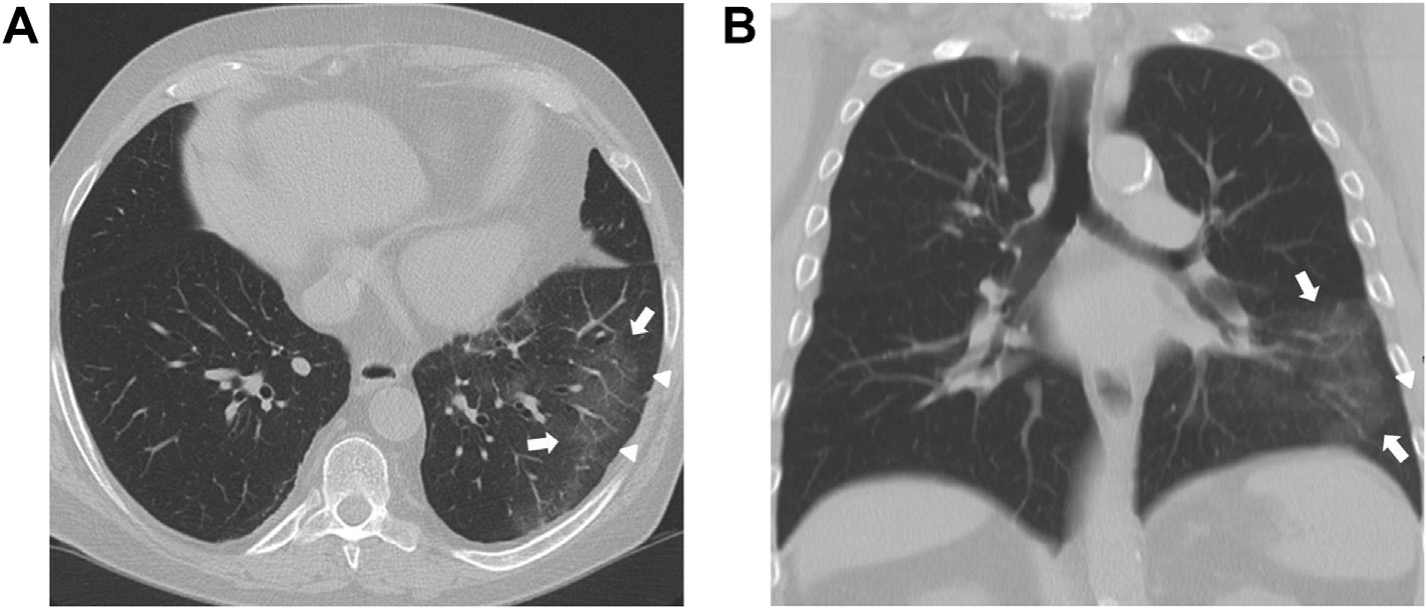
The initial CT scan acquired on the first day of hospitalization showed exclusive left-sided focal ground glass opacity (GGO) in the lower lobe (Figure 1B, coronal plane, *arrows*) with noticeable predominant exclusion of the subpleural space (Figure 1A, transverse plane, *arrowheads*). Associated inter- and intralobular septal thickening and bronchiectasis were present. No consolidation and no signs of lymphadenopathy or pleural effusion were found.

The initial CT scan obtained on the first day of hospitalization showed exclusive left-sided focal ground glass opacity (GGO) (Figure 1B) in the lower lobe with predominant exclusion of the subpleural space (Figure 1A). In some areas of GGO, slight inter- and intralobular septal thickening and bronchiectasis were detected.

**Figure 2:**
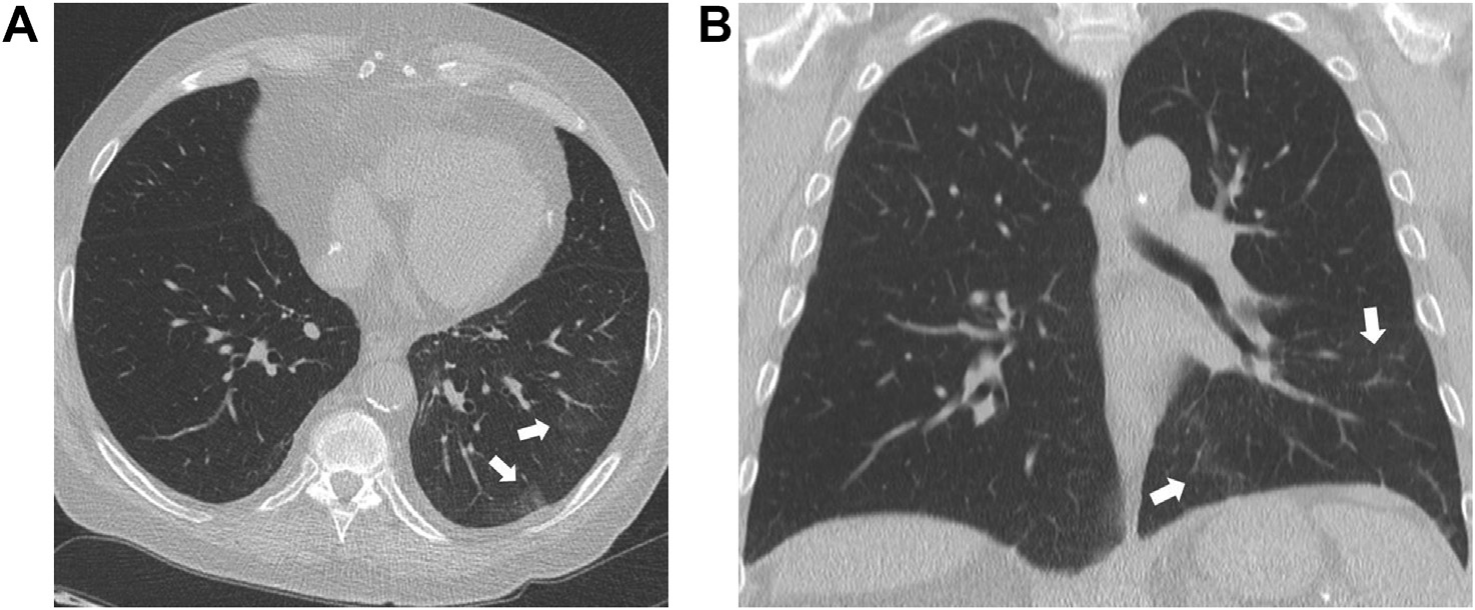
The second CT-scan on day 5 after hospitalization demonstrated already a significant decrease of ground glass opacities (GGO) in the left lower lobe (Figure 2A, transverse plane, *arrows*). No new GGO or consolidations were present compared to the initial CT scan. In addition, there was no pleural effusion or mediastinal lymphadenopathy (Figure 2B, coronal plane).

On day 5 of hospitalization, a second CT scan was performed. Although this timepoint is described as the point of peak levels of lung involvement in recent literature [7] the scan already showed a significant decrease of GGO in the left lower lobe with persistent inter- and intralobular septal thickening and bronchiectasis in some affected areas (Figure 2A and B).

**Figure 3:**
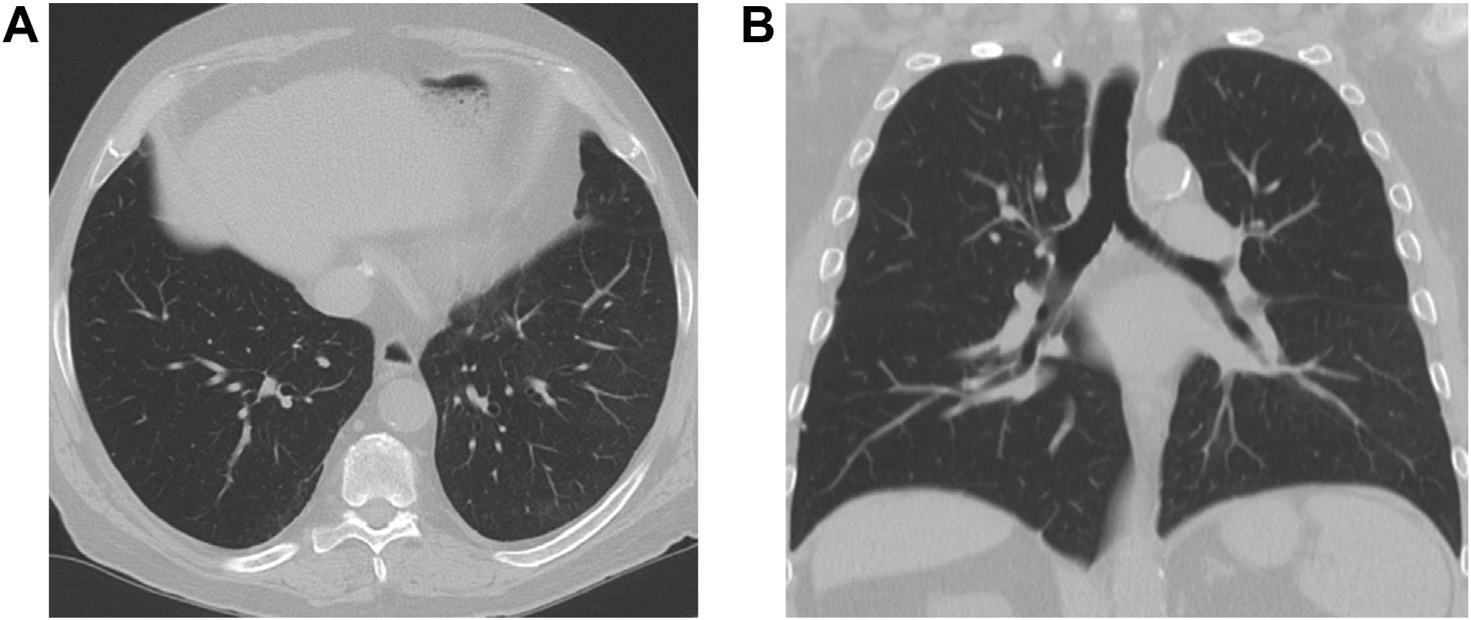
The final CT scan performed on the eight day of hospitalization showed only subtle residual ground glass opacities (GGO) in the left lower lobe (Figure 3A, transverse plane). Despite the GGO, no remaining structural lung changes in the context of pulmonary coronavirus disease 2019 (COVID-19) were present (Figure 3B, coronal plane).

Surprisingly, the third and final CT scan performed on day 8 of hospitalization showed only slight residual GGO in the left lower lobe (Figure 3A). Despite the GGO, no remaining structural lung changes in the context of COVID-19 were present (Figure 3B).

## 3 Patient discharge

After full recovery, the patient was discharged on hospitalization day 12 without any clinical symptoms and a negative RT-PCR test result for SARS-CoV-2.

## 4 Discussion

In the context of the global pandemic of COVID-19, for high risk patients [1, 8] with cardiovascular, pulmonary and immunological comorbidities (transplantation, immunotherapy, cancer), a severe course of illness is described with critical illness state of ARDS and high mortality rates [1]. Also, high age (>65 years) and male sex are considered as negative predictors. Patients fitting these attributes have a higher demand for ICU treatment and mechanical ventilation due to respiratory insufficiency [8, 9]. Due to long-term immunosuppressive medication after heart transplantation in 2003 and the presence of diabetes mellitus type II, the immune system of the reported patient was severely impaired. In addition, cardiovascular risk factors such as arterial hypertension, coronary 2-vessel disease, as well as a transplant vasculopathy and compromised renal function, made a critical course of COVID-19 more likely.

Against our expectations, the patient suffered from mild clinical symptoms throughout the duration of hospitalization. Over the course of three CT scans, we could observe an atypical course of COVID-19. Minimum intensity projections (MinIP) were read additionally, as it has been demonstrated in recent literature that they improve detectability of small GGOs [6]. Our patient developed unilateral left-sided GGO exclusively in the lower lobe, which already declined at day 5 of hospitalization and had subtotally disappeared at day 8 of hospitalization. The clinical course changed from initial mild dyspnea and hyperventilation with associated respiratory alkalosis in aBGA to barely any symptoms very quickly. Therefore, our patient was under ICU surveillance for only three days, where he had been initially taken as a high-risk patient. After being transferred to the normal ward, he showed no symptoms and could be discharged after a very short hospitalization time of only 12 days. Median time from illness onset to discharge from the hospital is 22 days according to a recent publication by Zhou et al. [1].

There have been descriptions of similarly mild courses, as seen in our patient [10, 11] with only mild stationary courses or even ambulant therapy in transplanted patients. But, there has also been reporting of lethal and critical cases [12]. In recent literature, patients with transplantation of the liver or kidney were reported as showing surprisingly mild courses of COVID-19 [3, 4, 13, 14]. In the largest study so far, Pereira et al reported on six mild and three severe courses for HTX recipients [15]. With a lethality of 18% the study reports a high risk for a severe course of disease in transplant patients. Corticosteroids and tacrolimus have been investigated for having protective effects on COVID-19 [16]. Less common immunosuppressive drugs, such as mycophenolate mofetil, are still being controversially discussed as they lack valid clinical data. Notably, none of the common immunosuppressive drugs such as Hydrocortisone and Tacrolimus have been proven to have a negative effect on patient outcome [5, 15]. So far it has been proven that high IL-6 peak levels are associated with severe clinical courses and poor outcome in patients suffering from COVID-19 [16]. A similar finding by Gao et al. reported that IL-6 and d-dimer levels were significantly related to the severity of COVID-19 [17]. This may be explained by the pathways related to pro-inflammatory cytokines, such as interleukin IL-1b and IL-6. SARS-CoV-2 is assumed to bind to Toll-like receptors (TLR). This leads to the release of pro-IL-1b causing activation of a cascade of active IL-1b which represents a mediator of severe lung inflammation (which may result in a ARDS), fever and fibrosis [16, 18, 19]. Therefore, it is conceivable that low IL-1b and IL-6 levels, which are present in patients receiving immunosuppressive therapy, may play a role for prevention of a severe course of COVID-19.

From our observations we conclude that not every patient with immunosuppressive therapy and multiple risk factors suffers from a severe course of COVID-19.

We therefore hypothesize, that patients with HTX and other risk factors may benefit from the combination of persistent anticoagulative and immunosuppressive therapy in the context of COVID-19.

## Declarations

### Author contribution statement

All authors listed have significantly contributed to the investigation, development and writing of this article.

### Funding statement

This research did not receive any specific grant from funding agencies in the public, commercial, or not-for-profit sectors.

### Data availability statement

Data included in article/supplementary material/referenced in article.

### Declaration of interests statement

The authors declare no conflict of interest.

### Additional information

No additional information is available for this paper.
